# Hypoxia-Inducible Factor-1*α* Knockdown Plus Glutamine Supplementation Attenuates the Predominance of Necrosis over Apoptosis by Relieving Cellular Energy Stress in Acute Pancreatitis

**DOI:** 10.1155/2019/4363672

**Published:** 2019-06-02

**Authors:** Liang Ji, Xiaoyu Guo, Jiachen Lv, Fan Xiao, Wangjun Zhang, Jie Li, Zhitao Lin, Bei Sun, Gang Wang

**Affiliations:** ^1^The Department of General Surgery, The No. 1 Affiliated Hospital of Harbin Medical University, Harbin, 150001 Heilongjiang Province, China; ^2^The Department of General Surgery, The No. 3 Affiliated Hospital of Harbin Medical University, Harbin, 150001 Heilongjiang Province, China

## Abstract

The present study was conducted to investigate the effect and potential mechanism of hypoxia-inducible factor-1*α* (HIF-1*α*) genetic inhibition plus glutamine (Gln) supplementation on necrosis-apoptosis imbalance during acute pancreatitis (AP), with a specific focus on the regulations of intracellular energy metabolism status. Wistar rats and AR42J cells were used to establish AP models. When indicated, a HIF-1*α* knockdown with or without a Gln supplementation was administered. *In vivo*, local and systemic inflammatory injuries were assessed by serum cytokine measurement, H&E staining, and transmission electron microscope (TEM) observation of pancreatic tissue. *In vitro*, intracellular energy metabolism status was evaluated by measuring the intracellular adenosine triphosphate (ATP), lactic acid, and Ca^2+^ concentrations and the mitochondrial potential. In addition, changes in the apoptotic activity were analyzed using TUNEL staining *in vivo* and an apoptosis assay *in vitro.* HIF-1*α* knockdown alleviated AP-related inflammatory injury as indicated by the measurements of serum cytokines and examinations of TEM and H&E staining of pancreatic tissues. HIF-1*α* knockdown played an antioxidative role against AP-related injuries by preventing the increase in the intracellular Ca^2+^ concentration and the decrease in the mitochondrial membrane potential and subsequently by suppressing the glycolysis pathway and increasing energy anabolism in AR42J cells after AP induction. Apoptosis was significantly upregulated when HIF-1*α* was knocked down before AP induction due to an attenuation of the translocation of nuclear factor-kappa B to the nuclei. Furthermore, these merits of HIF-1*α* knockdown in the relief of the metabolic stress and upregulation of apoptosis were more significant when Gln was administered concomitantly. In conclusion, Gln-supplemented HIF-1*α* knockdown might be promising for the future management of AP by relieving the intracellular energy stress, thereby attenuating the predominance of necrosis over apoptosis.

## 1. Introduction

Acute pancreatitis (AP) may present as a mild self-limiting condition or as a lethal gastrointestinal disorder. The mechanisms of AP remain elusive, and no specific cure has yet been developed [1–6]. The progression and prognosis of AP mainly depend on the dominant death mode of the acinar cells. During the natural course of AP, the injured acinar cells are directed towards two major cell death pathways, namely, necrosis and apoptosis. Unlike apoptosis which acts as a self-defense mechanism against AP-related injuries, necrosis usually correlates positively with the severity of AP [7]. Therefore, relieving the predominance of necrosis over apoptosis by attenuating the necrosis and/or promoting apoptosis is a promising direction in the management of AP.

Recent studies have indicated that apoptosis and necrosis of pancreatic acinar cells during AP are interconvertible rather than being completely fixed under certain circumstances. The level of adenosine triphosphate (ATP), an index of intracellular energy metabolism, is pivotal in regulating the switching and interactions between apoptosis and necrosis [8]. Stress might induce overconsumption of intracellular ATP, which would facilitate necrosis. However, high levels of ATP tend to upregulate cellular apoptosis [9]. Thus, numerous studies have been conducted to investigate the role of energy metabolism in the pathogenesis of AP. Up to the present, there have been no reports regarding the interconversion of cellular necrosis-apoptosis through the regulation of energy metabolism status in AP.

Hypoxia-inducible factor-1 (HIF-1), a unique regulatory transcriptional factor which can be activated and highly expressed in response to hypoxia, has gained a worldwide attention due to its dominant role in the regulation of intracellular energy metabolism in some inflammatory and immune diseases. HIF-1 is a heterodimer composed of *α* and *β* subunits. Unlike HIF-1*β*, which demonstrates stable intracellular expression, HIF-1*α* is regulated by the incubated oxygen exposure concentration and acts as the major active unit of HIF-1 [10, 11]. By regulating the transcription and expression of target genes, HIF-1*α* has the inverse effects of shutting down the tricarboxylic acid cycle and facilitating the glycolysis pathway. These processes inhibit the ATP production and activate the inflammatory response [12, 13]. Gomez et al. [14] found that 8-12 hours after establishing a mouse model of AP, the level of HIF-1*α* in the pancreatic tissue was significantly increased.

Metabolically, glutamine (Gln) has long been known to be a nonessential amino acid. However, it serves as a conditionally essential amino acid in response to stress and injury [15]. Gln is a source of fuel for lymphocytes and enterocytes and might play an antioxidative role as a precursor for glutathione and a cytoprotective role by upregulating heat shock proteins [16]. It has been shown that Gln supplementation during AP maintains gut integrity, improves the immune response, and decreases the release of some proinflammatory mediators by inhibiting the activation of nuclear factor-kappa B (NF-*κ*B) and p38 mitogen-activated protein kinase [17–19]. On the basis of the solubility and instability of Gln, L-alanyl-L-glutamine is usually administered as a donor *in vivo* because it is a dipeptide that dissociates into free Gln soon after entry into the circulatory system [20].

We therefore performed the present study to investigate the effect and potential mechanism of HIF-1*α* genetic inhibition plus Gln supplementation on necrosis-apoptosis imbalance during AP with a specific focus on the regulation of intracellular energy metabolism status.

## 2. Materials and Methods

### 2.1. Animals and Reagents

A total of 60 male Wistar rats (200-220 g) were supplied by the Animal Research Center, the First Clinical College of Harbin Medical University (Harbin, China). The rats were fed with rodent chow and water *ad libitum* in an environmentally controlled room (18-21°C, 40-60% relative humidity, and 12 h light/dark cycle). After an acclimatization for one week, the rats were fasted overnight before the experiments. All surgical procedures and cares administered to the rats were approved by the Institutional Animal Care and Use Committee of Harbin Medical University (No. 2011BS001).

Sodium taurocholate (Na-TC) and L-alanyl-L-glutamine were purchased from Sigma-Aldrich (St. Louis, MO, USA). A commercial lentivirus-mediated small interfering RNA (siRNA) kit was purchased from Genecoopia (San Francisco, MO, USA) to silence HIF-1*α*. Antibodies used in the present study are cleaved PARP (Asp214), cleaved caspase-3 (Asp175), cleaved caspase-9 (Asp353), and nuclear factor-*κ*B p65 purchased from Cell Signaling Technology (Danvers, MA, USA); *β*-actin purchased from Santa Cruz Biotechnology (Santa Cruz, CA, USA); and HIF-1*α* purchased from Novus (Littleton, CO, USA).

### 2.2. Experimental Design *In Vivo*

A model of AP in rats was induced as described previously [21]. Briefly, the rats were anesthetized by an intraperitoneal injection of sodium pentobarbital (30 mg/kg). Then, a midline laparotomy was performed and the distal pancreaticobiliary duct was ligated. AP was induced by a retrograde infusion of 3.5% Na-TC (0.15 mL/100 g) into the pancreaticobiliary duct.

The rats were allocated into four groups (*n* = 10): sham, AP, HIF-1*α* siRNA, and HIF-1*α* siRNA+Gln groups. AP was induced in the rats in AP, HIF-1*α* siRNA, and HIF-1*α* siRNA+Gln groups, whereas the rats in the sham group were subjected only to laparotomy. Based on our preliminary experiments, an injection of lentivirus-mediated HIF-1*α* siRNA (Lv-siHIF-1*α*) particles [10^6^ TU/mL in 200 *μ*L of phosphate-buffered saline (PBS)] through the caudal vein 5 d before the experiment was performed to silence HIF-1*α* in rats. In sham and AP groups, an equivalent level of negative control (Lv-siNC) particles was administered instead. Additionally, the rats in the HIF-1*α* siRNA+Gln group received an intravenous injection of L-alanyl-L-glutamine (0.4 g/kg) through the caudal vein immediately after AP induction [18, 22, 23]. The rats were sacrificed at 24 h after AP induction, and blood and pancreatic samples were collected. The serum was obtained after a centrifugation at 3000 rpm for 15 min and then stored at -80°C until assay. The pancreatic tissue was prepared for three different purposes: (1) rinsed in saline buffer and snap-frozen in liquid nitrogen at -80°C for Western blot, (2) fixed in 4% buffered paraformaldehyde for 48 h and then embedded in paraffin for hematoxylin-eosin (H&E) staining and terminal deoxynucleotidyl transferase-mediated dUTP nick end labeling (TUNEL) staining, and (3) fixed in 2 mL of 2.5% glutaraldehyde and then postfixed in 1% osmium tetroxide solution for transmission electron microscopy (TEM).

### 2.3. Measurement of Parameters in Serum and Pancreas

The serum levels of amylase and C-reactive protein (CRP) were spectrophotometrically measured using a biochemical autoanalyzer (Toshiba, Japan) as previously described [24]. The serum and pancreatic levels of tumor necrosis factor-*α* (TNF-*α*) and interleukin-1*β* (IL-1*β*) were measured using enzyme-linked immunosorbent assay kits (R&D Systems, Minneapolis, MN, USA) according to the manufacturer's instructions.

### 2.4. H&E Staining

H&E staining was performed to examine the level of inflammation and tissue damage under a light microscope (40x). Two experienced pathologists who were blinded to the experimental protocol scored the pancreatic tissue on a scale from 0 to 4 for the degrees of edema, inflammation, hemorrhage, and necrosis respectively in 20 randomized high-power fields according to the histological scoring criteria as Kusske et al. reported [25]. Then, the final score was totaled for each group.

### 2.5. TEM

The fixed samples were dehydrated through a graded series of ethanol and embedded in epoxy resin. Ultrathin sections (80 nm) were collected on copper grids, double-stained with uranyl acetate and lead citrate, and then examined under a Hitachi H-7100 transmission electron microscope (Hitachinaka, Japan) at 80 kV.

### 2.6. Measurement of Myeloperoxidase (MPO) and Lipid Peroxidase (LPO) in the Pancreas

The pancreatic levels of MPO and LPO were measured with the specific kits (Jiancheng Bioengineering Institute, Nanjing, China) according to the manufacturer's instructions. The former was expressed as units per gram of pancreatic homogenates, and the latter was expressed as micromoles per gram of pancreatic homogenates.

### 2.7. TUNEL Staining

The apoptosis of pancreatic acinar cells was determined using a TUNEL Apoptosis Detection Kit (Beyotime Biotechnology, Shanghai, China) according to the manufacturer's instructions [26]. Cellular nuclei with the presence of a dark reddish-brown chromogen were identified as positive. The TUNEL-positive cells were counted in 10 randomly selected high-power fields under a light microscope and expressed as a percentage of the total cell counts (apoptosis index).

### 2.8. Electrophoretic Mobility Shift Assay (EMSA)

A nuclear extraction kit and an EMSA kit were purchased from Thermo Fisher Scientific (Rockford, IL, USA) to determine the DNA binding activities of NF-*κ*B and HIF-1*α*. Briefly, 2 *μ*L of nuclear extract (at a concentration of 2 *μ*g/*μ*L) was incubated with 1 *μ*L of biotin-labeled probe containing the NF-*κ*B- (5′-AGTTGAGGGGACTTTCCCAGGC-3′) or HIF-1*α*- (5′-TCTGTACGTGACCACACTCACCTC-3′) binding domain for 30 min at 15°C. The reaction mixtures were separated on 6% nondenaturing polyacrylamide gels in 0.5x *tris*-borate ethylenediamine tetraacetic acid at 120 V for 60 min at 4°C and transferred onto a presoaked membrane at 300 mA for 30 min at 4°C. The membranes were subjected to ultraviolet light-induced cross-linking for 3 min and then incubated with blocking buffer containing a stabilized streptavidin-horseradish peroxidase conjugate (1 : 1 000) for 30 min. The bands on the membranes were detected with a Chemiluminescent Nucleic Acid Detection Module Kit (Thermo Fisher Scientific).

### 2.9. Cell Cultures

The rats pancreatic exocrine cell line AR42J was purchased from the American Type Culture Collection (Manassas, VA, USA) and cultured in Dulbecco's modified Eagle's medium (Gibco, Grand Island, NY, USA) supplemented with 10% fetal bovine serum (ScienCell, San Diego, CA, USA), 100 U/mL of penicillin, and 100 mg/mL of streptomycin (Invitrogen, Carlsbad, CA, USA) at 37°C in a 5% CO_2_-humidified incubator.

### 2.10. Experimental Design *In Vitro*

There were four groups *in vitro*: control, AP, HIF-1*α* siRNA, and HIF-1*α* siRNA+Gln groups. AR42J cells in control and AP groups were transfected with Lv-siNC particles whereas the counterparts in the HIF-1*α* siRNA and HIF-1*α* siRNA+Gln groups were transfected with Lv-siHIF-1*α*. AR42J cells were plated in 6-well plates (5 × 10^4^ per well) until 70% confluent so that appropriate volumes of lentivirus could be added to achieve the multiplicity of the infection value recommended by the manufacturer. To simulate AP, the Lv-siHIF-1*α* or Lv-siNC particle-pretreated cells were incubated with 500 *μ*M of Na-TC for 12 h [27, 28]. When indicated, L-alanyl-L-glutamine (2 mM) was administered together with AP stimulation [29, 30]. The culture medium supernatants were collected to evaluate lactate dehydrogenase (LDH) release and glucose uptake with a biochemical autoanalyzer (Toshiba, Tokyo, Japan) as previously described [24].

### 2.11. Measurements of Intracellular ATP and Lactic Acid

The intracellular levels of ATP and lactic acid were measured with specific kits (Jiancheng Bioengineering Institute) according to the manufacturer's instructions and then normalized to the protein concentrations, which were determined using the bicinchoninic acid method. The former was expressed as nmol/mg, and the latter was expressed as mmol/g.

### 2.12. Measurement of Intracellular Ca^2+^ Concentration

The method for the measurement of intracellular Ca^2+^ concentration has been described previously [31]. In brief, the cells were preloaded with 5 *μ*M of Fura-2 AM (Beyotime, Shanghai, China) in HEPES buffer for 1 h at room temperature. Images of the Fura-2-loaded cells were captured using a laser confocal microscope (LSM 510, Carl Zeiss, Oberkochen, Germany) and analyzed using Image-Pro Plus v6.0 software (Media Cybernetics, Crofton, MA, USA). Background-subtracted fluorescent images for excitation at 340 nm and 380 nm were captured. The intracellular Ca^2+^ concentration was estimated from the ratio of Fura-2 fluorescence emitted at 510 nm after excitation at 340 nm to that after excitation at 380 nm, according to the Grynkiewicz equation [32].

### 2.13. Intracellular Mitochondrial Potential

Intracellular mitochondrial potential (MP) was determined using a dual-emission mitochondrial dye 5,5′,6,6′-tetrachloro-1,1′,3,3′-tetraethylben-zimidazolocarbocyanine iodide (JC-1, Beyotime) as detailed elsewhere [33]. In short, staining was performed using 2.5 *μ*g/mL of JC-1 for 15 min at 37°C. After staining, the cells were rinsed 3 times with PBS. Dye equilibration was allowed for 10 min at room temperature prior to imaging. Fluorescent images of the emissions at 529 nm and 590 nm were captured using a laser confocal microscope (Carl Zeiss). JC-1 exhibits a fluorescence emission shift upon aggregation from 529 nm (green monomer, indicative of low MP) to 590 nm (red “J-aggregates,” indicative of high MP). Thus, a reduced ratio of red/green fluorescence indicates mitochondrial depolarization.

### 2.14. Apoptosis Assay

Apoptosis was determined by Annexin V and propidium iodide staining (BD Biosciences, Shanghai, China) as described previously [24]. Theoretically, Annexin V and propidium iodide double-staining indicates necrosis whereas Annexin V single-staining indicates apoptosis. The images were acquired using a confocal laser scanning microscope (Carl Zeiss), and the average percentages of apoptotic cells were calculated in 5 randomly selected high-power fields.

### 2.15. Western Blot Analysis

The protocol for Western blot has been described previously [24, 27, 28]. In brief, pancreatic tissue or cells were homogenized in a protein lysis buffer (Beyotime, Shanghai, China) that contained the protease inhibitor (Roche) and phosphatase inhibitor (Roche) and centrifuged at 10 000g for 10 min at 4°C. The samples containing 50 *μ*g of total protein were separated using 10% polyacrylamide SDS gels and electrophoretically transferred to polyvinylidene difluoride membranes. The membranes were blocked with 5% skim milk then incubated with appropriate primary antibodies (dilution for all: 1 : 1000) and horseradish peroxidase-conjugated secondary antibodies. The immunostained protein bands were detected using an enhanced chemiluminescence kit (Pierce Chemical, Rockford, IL, USA). *β*-Actin was used as the protein loading control, and the level of protein expression was corrected using the band density relative to that of *β*-actin.

### 2.16. Statistical Analysis

The data are presented as the mean ± standard deviation (SD) of three independent experiments and analyzed using SAS 9.1 for Windows (SAS Institute, Cary, NC, USA). Comparisons between multiple groups were performed using a one-way ANOVA followed by Dunnett's *t*-test. A *P* value of <0.05 was taken to be statistically significant.

## 3. Results

### 3.1. The Upregulation of HIF-1*α* after AP Induction Was Prevented by Prior HIF-1*α* Knockdown

**Figure 1:**
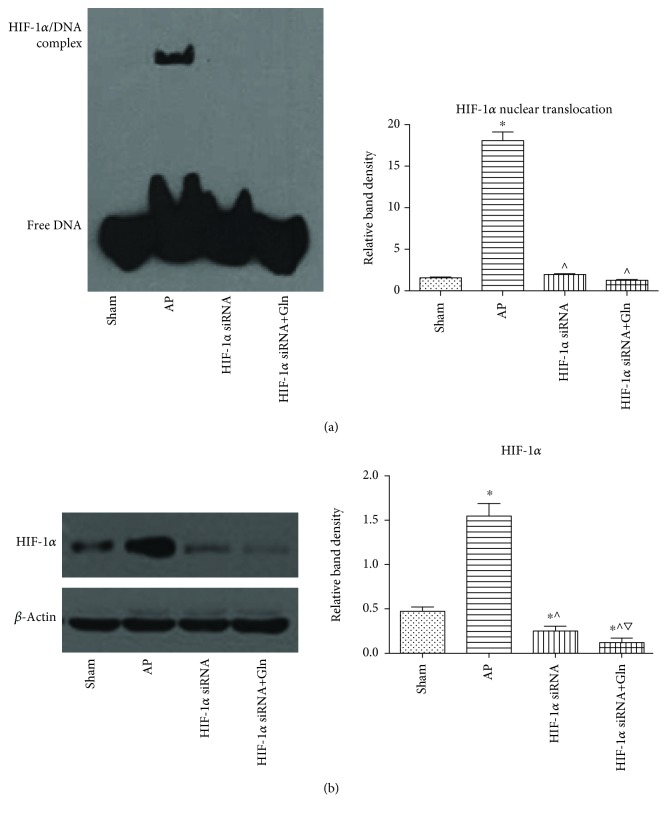
The upregulation of HIF-1*α* after AP induction was prevented by prior HIF-1*α* knockdown. (a) Representative EMSA blots (left) and quantifications (right) of HIF-1*α* DNA-biding activity in pancreatic tissues harvested from the rats that were subjected to sham operation, AP, HIF-1*α* siRNA, and HIF-1*α* siRNA+Gln for 24 h since AP induction. (b) Representative immunoblot images (left) and quantifications (right) of HIF-1*α* protein expression in pancreatic tissues harvested from the rats as described above. *β*-Actin was used as the protein loading control. Data were presented as mean ± SD (*n* = 3). ^∗^*P* < 0.05*versus* sham, ^^^*P* < 0.05*versus* AP, and ^▽^*P* < 0.05*versus* HIF-1*α* siRNA. Abbreviations: AP: acute pancreatitis; EMSA: electrophoretic mobility shift assay; Gln: glutamine; HIF-1*α*: hypoxia inducible factor-1*α*; SD: standard deviation.

As Figure 1(a) shows, the HIF-1*α*-DNA binding activity was significantly increased in the AP group compared to that of the sham group. However, the HIF-1*α* DNA-binding activity associated with AP induction was significantly reduced by prior HIF-1*α* knockdown. The detectable levels of HIF-1*α* expression among these groups also confirmed these results (Figure 1(b)). That is, the level of HIF-1*α* expression was increased by AP induction and this increase was significantly inhibited when HIF-1*α* was knocked down before AP induction.

### 3.2. HIF-1*α* Knockdown Alleviated AP-Related Inflammatory Injury, and This Therapeutic Effect Was Enhanced by the Additional Administration of Gln

**Figure 2:**
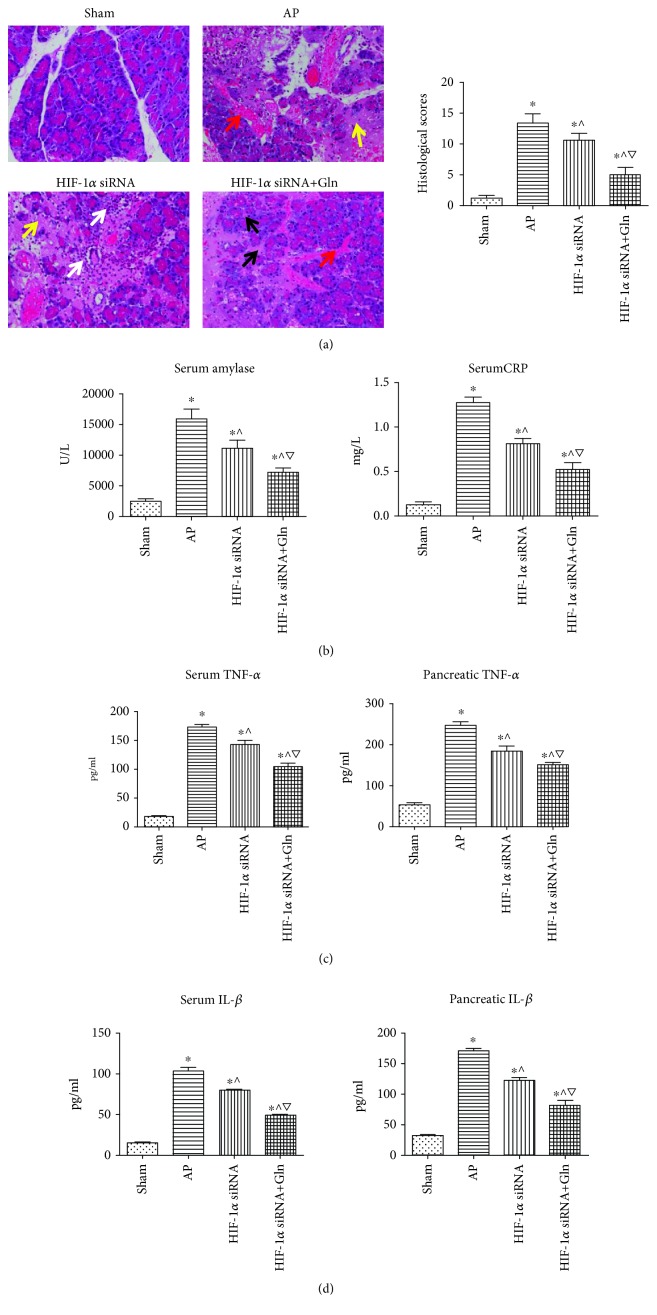
HIF-1*α* knockdown alleviated AP-related inflammatory injury, and this therapeutic effect was enhanced by the additional administration of Gln. (a) Representative photos (40x) and histological scores of H&E-stained pancreatic tissues harvested from the rats as described in Figure 1(a). Black arrow indicates edema, white arrow indicates inflammatory infiltration, yellow arrow indicates necrosis, and red arrow indicated hemorrhage. (b) The serum levels of amylase and CRP in rats as described in Figure 1(a). (c) The serum (left) and pancreatic (right) levels of TNF-*α* in rats as described in Figure 1(a). (d) The serum (left) and pancreatic (right) levels of IL-1*β* in rats as described in Figure 1(a). Data were presented as mean ± SD (*n* = 3). ^∗^*P* < 0.05*versus* sham, ^^^*P* < 0.05*versus* AP, and ^▽^*P* < 0.05*versus* HIF-1*α* siRNA. Abbreviations: AP: acute pancreatitis; CRP: C-reactive protein; Gln: glutamine; H&E: hematoxylin-eosin; HIF-1*α*: hypoxia inducible factor-1*α*; IL-1*β*: interleukin-1*β*; SD: standard deviation; TNF-*α*: tumor necrosis factor-*α*.

AP induction, with or without any intervention, was associated with a range of levels of edema, necrosis, inflammatory cell infiltration, and hemorrhage whereas there was no evident ectopic in rats that had undergone a sham operation. The histological scoring indicated that the morphological alterations were most severe in the AP group, followed by the HIF-1*α* siRNA group and HIF-1*α* siRNA+Gln group (Figure 2(a)). The same results were obtained for the measurements of the serum levels of amylase and CRP (Figure 2(b)) as well as the levels of TNF-*α* and IL-1*β* both in sera and pancreatic tissues (Figures 2(c) and 2(d)). Thus, these results indicated that HIF-1*α* knockdown alleviated AP-related inflammatory injures, and this therapeutic effect was further enhanced by the additional administration of Gln.

### 3.3. HIF-1*α* Silencing Played an Antioxidative Role against AP-Related Injuries, and This Effect Was Enhanced by the Additional Administration of Gln

**Figure 3:**
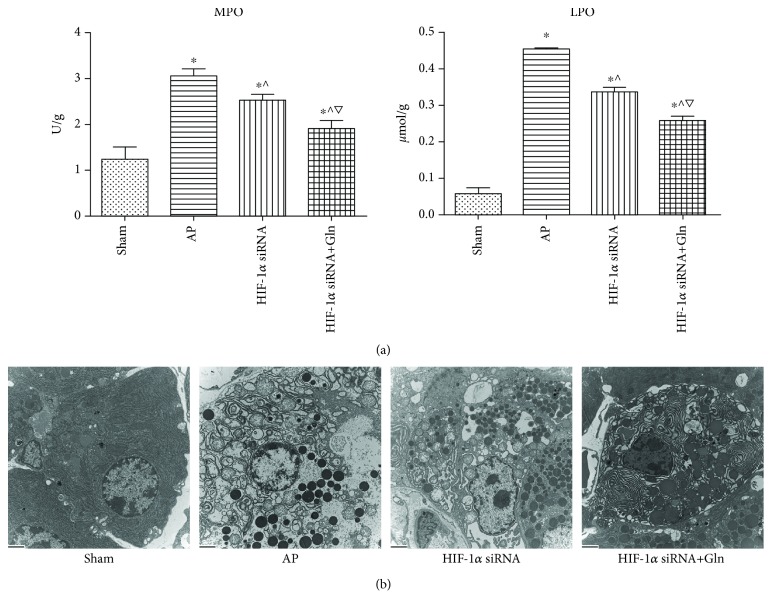
HIF-1*α* silencing played an antioxidative role against AP-related injuries, and this effect was enhanced by the additional administration of Gln. (a) The pancreatic levels of MPO (left) and LPO (right) in rats as described in Figure 1(a). (b) Representative TEM photos of pancreatic tissues harvested from the rats as described in Figure 1(a), bar = 2 *μ*m. Data were presented as mean ± SD (*n* = 3). ^∗^*P* < 0.05*versus* sham, ^^^*P* < 0.05*versus* AP, and ^▽^*P* < 0.05*versus* HIF-1*α* siRNA. Abbreviations: AP: acute pancreatitis; Gln: glutamine; HIF-1*α*: hypoxia-inducible factor-1*α*; LPO: lipid peroxidase; MPO: myeloperoxidase; SD: standard deviation; TEM: transmission electron microscope.

To evaluate the levels of oxidative stress response among the animals treated with the various procedures, the pancreatic levels of MPO and LPO were measured. AP induction was significantly associated with increases in the levels of both MPO and LPO compared to those in the sham group. HIF-1*α* silencing before AP induction significantly decreased the levels of MPO and LPO when compared with those in the AP group. Moreover, the levels of MPO and LPO in the HIF-1*α* siRNA+Gln group were significantly decreased compared to those in the HIF-1*α* siRNA group (Figure 3(a)). Such effects on the oxidative stress response due to HIF-1*α* silencing, with or without Gln supplementation before AP induction, were also suggested by the ultrastructural alterations that were observed using TEM. As shown in Figure 3(b), AP induction was associated with mitochondrial swelling, endoplasmic reticulum disorder, and nuclear fragmentation. Notably, these ultrastructural alterations were most severe in the AP group, followed by those in the HIF-1*α* siRNA group and those in the HIF-1*α* siRNA+Gln group.

### 3.4. Apoptosis Was Significantly Upregulated when HIF-1*α* Knockdown Was Administered before AP Induction, and This Effect Was Enhanced by a Concurrent Administration of Gln

**Figure 4:**
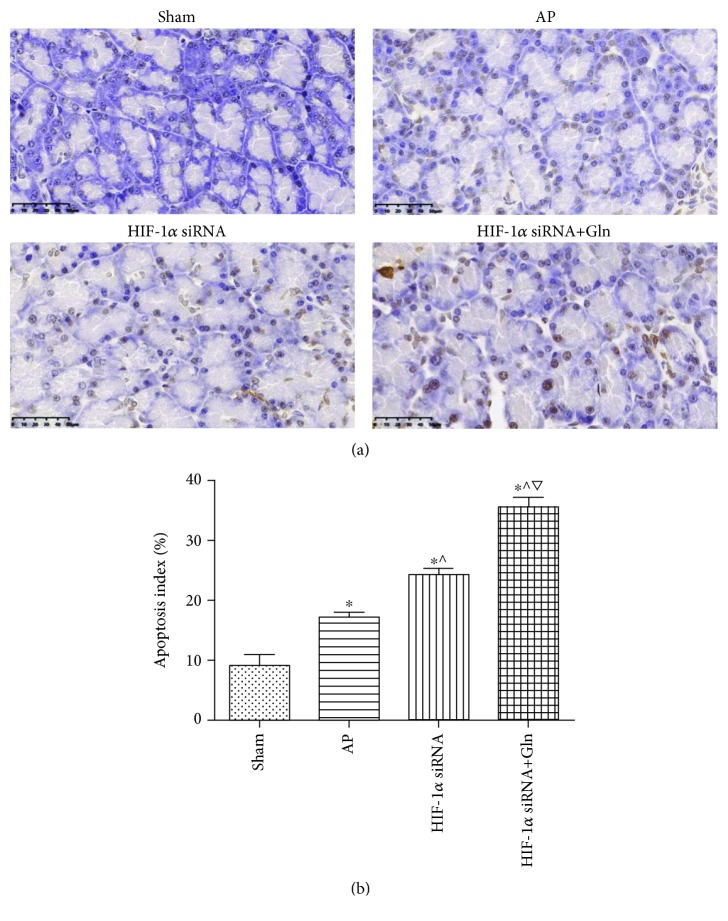
Apoptosis was significantly upregulated when HIF-1*α* knockdown was administered before AP induction, and this effect was enhanced by a concurrent administration of Gln. (a) Representative TUNEL photos of pancreatic tissues harvested from the rats as described in Figure 1(a), bar = 50 *μ*m. (b) The ratio of TUNEL-positive cell counts to total cell counts was calculated to yield an apoptosis index in each group. Data were presented as mean ± SD (*n* = 3). ^∗^*P* < 0.05*versus* sham, ^^^*P* < 0.05*versus* AP, and ^▽^*P* < 0.05*versus* HIF-1*α* siRNA. Abbreviations: AP: acute pancreatitis; Gln: glutamine; HIF-1*α*: hypoxia inducible factor-1*α*; SD: standard deviation; TUNEL: terminal deoxynucleotidyl transferase-mediated dUTP nick end labeling.

TUNEL staining of the pancreatic sections was observed under a light microscope (Figure 4(a)). The results showed that HIF-1*α* knockdown before AP induction increased the apoptosis index compared with that in the AP group. Moreover, a concurrent administration of Gln with HIF-1*α* knockdown significantly increased the apoptosis index than that in the HIF-1*α* knockdown group (Figure 4(b)).

### 3.5. HIF-1*α* Knockdown Attenuated NF-*κ*B Nuclear Translocation Induced by AP, and This Effect Was Enhanced by a Concurrent Administration of Gln

**Figure 5:**
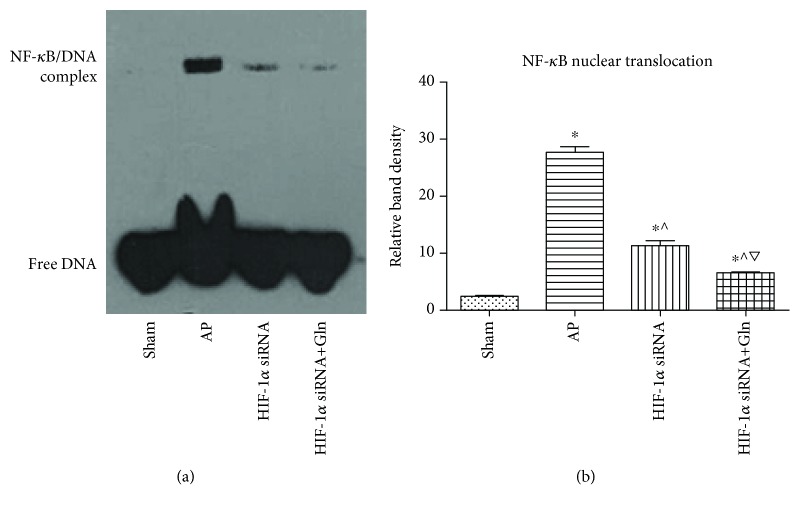
HIF-1*α* knockdown attenuated NF-*κ*B nuclear translocation induced by AP, and this effect was enhanced by a concurrent administration of Gln. (a and b) Representative EMSA blots (a) and quantifications (b) of NF-*κ*B DNA-binding activity in pancreatic tissues harvested from the rats as described in Figure 1(a). Data were presented as mean ± SD (*n* = 3). ^∗^*P* < 0.05*versus* sham, ^^^*P* < 0.05*versus* AP, and ^▽^*P* < 0.05*versus* HIF-1*α* siRNA. Abbreviations: AP: acute pancreatitis; EMSA: electrophoretic mobility shift assay; Gln: glutamine; HIF-1*α*: hypoxia-inducible factor-1*α*; NF-*κ*B: nuclear factor-kappa B; SD: standard deviation.

As the most important regulator in the pathogenesis of AP, the activation of NF-*κ*B might upregulate the expression of proinflammatory factors, antiapoptotic proteins, and other important mediators that account for the local and systemic responses to AP induction. It was shown that the nuclear translocation of HIF-1*α* was significantly increased by AP induction compared to that in the sham group (Figure 1(a)). Moreover, HIF-1*α* knockdown before AP induction significantly ameliorated the level of NF-*κ*B nuclear translocation compared with that induced by AP induction alone. In addition, the level of NF-*κ*B nuclear translocation was significantly lower in the HIF-1*α* siRNA+Gln group than that in the HIF-1*α* siRNA group (Figures 5(a) and 5(b)).

### 3.6. HIF-1*α* Knockdown Suppressed the Glycolysis Pathway and Increased Energy Anabolism in AR42J Cells, and These Effects Were Enhanced when Gln Was Administered Concomitantly

**Figure 6:**
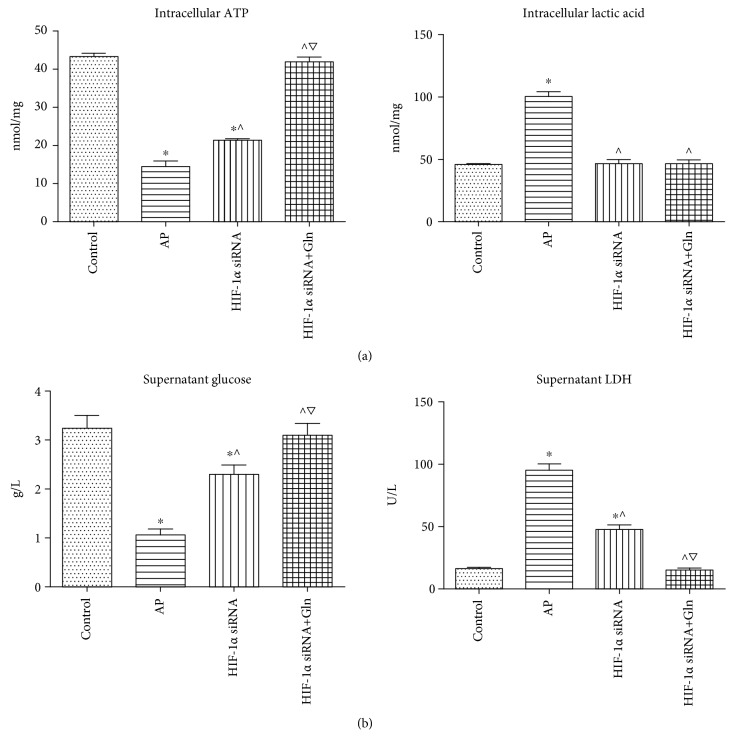
HIF-1*α* knockdown suppressed the glycolysis pathway and increased energy anabolism in AR42J cells, and these effects were more profound if Gln was administered concomitantly. (a) The intracellular levels of ATP (left) and lactic acid (right) in AR42J cells that were subjected to control, AP, HIF-1*α* siRNA, and HIF-1*α* siRNA+Gln for 12 h since AP simulation. (b) The supernatant levels of glucose (left) and LDH (right) when AR42J cells were subjected to control, AP, HIF-1*α* siRNA, and HIF-1*α* siRNA+Gln for 12 h since AP simulation. Data were presented as mean ± SD (*n* = 3). ^∗^*P* < 0.05*versus* sham, ^^^*P* < 0.05*versus* AP, and ^▽^*P* < 0.05*versus* HIF-1*α* siRNA. Abbreviations: AP: acute pancreatitis; ATP: adenosine triphosphate; Gln: glutamine; HIF-1*α*: hypoxia-inducible factor-1*α*; LDH: lactate dehydrogenase; SD: standard deviation.

After HIF-1*α* silencing, the levels of ATP and lactic acid in each group were measured (Figure 6(a)). AP induction was significantly associated with a decrease in ATP level and an increase in lactic acid level compared with those in the control group. HIF-1*α* silencing, which was conducted before AP induction, was significantly associated with an increase in ATP level and a decrease in lactic acid level compared with those in the AP group. In addition, a concomitant administration of Gln with HIF-1*α* silencing was significantly correlated with an increase in ATP level compared with that in the HIF-1*α* siRNA group. In accordance with previous intracellular findings, the measurement of supernatant glucose and LDH also indicated that HIF-1*α* knockdown suppressed the glycolysis pathway and increased energy anabolism in AR42J cells, and these effects were enhanced when Gln was administered concomitantly (Figure 6(b)).

### 3.7. HIF-1*α* Knockdown Prevented the Increase in the Intracellular Ca^2+^ Concentration and the Decrease in the Mitochondrial Membrane Potential in AR42J Cells after AP Induction, and These Effects Were Enhanced when Gln Was Administered Concomitantly

**Figure 7:**
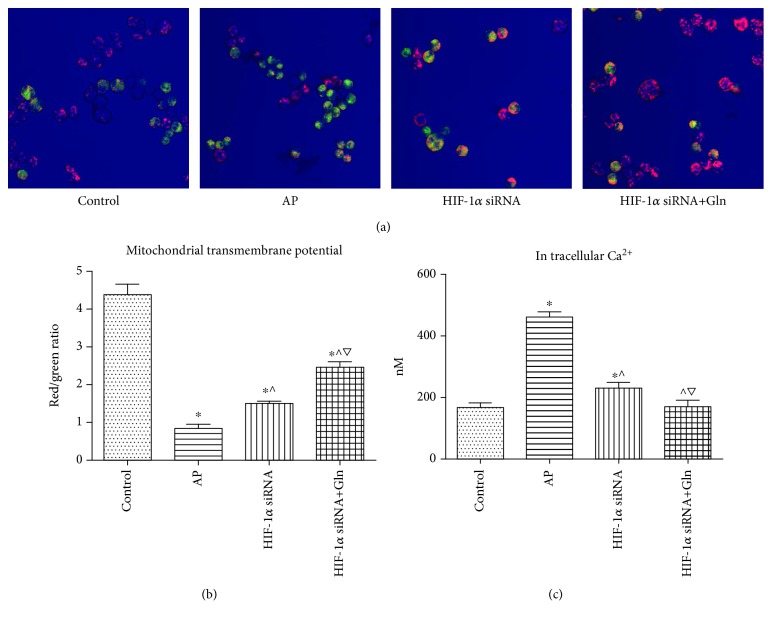
HIF-1*α* knockdown prevented the increase in the intracellular Ca^2+^ concentration and the decrease in the mitochondrial membrane potential in AR42J cells after AP induction, and these effects were enhanced when Gln was administered concomitantly. (a and b) Representative fluorescent images (a, 20x) indicative of intracellular mitochondrial potential by JC-1 staining in AR42J cells as described in Figure 6(a), and the ratio of red/green fluorescence (b) was calculated to indicate intracellular mitochondrial potential. (c) The intracellular concentration of Ca^2+^ in AR42J as described in Figure 6(a). Data were presented as mean ± SD (*n* = 3). ^∗^*P* < 0.05*versus* sham, ^^^*P* < 0.05*versus* AP, and ^▽^*P* < 0.05*versus* HIF-1*α* siRNA. Abbreviations: AP: acute pancreatitis; Gln: glutamine; HIF-1*α*: hypoxia-inducible factor-1*α*; SD: standard deviation.

As shown in Figures 7(a) and 7(b), a certain degree of mitochondrial depolarization developed after AP induction was indicated by the significant decreased red/green fluorescence ratio compared to that in the control group. HIF-1*α* knockdown before AP induction significantly corrected the mitochondrial membrane potential that resulted from AP induction alone, and this effect was enhanced when Gln supplementation was administered together with HIF-1*α* knockdown. In addition, a prior HIF-1*α* knockdown corrected the increase in the intracellular Ca^2+^ concentration that resulted from AP induction alone, and this effect was enhanced when Gln was administered concomitantly (Figure 7(c)).

### 3.8. HIF-1*α* Knockdown Decreased Necrosis and Increased Apoptosis within AR42J Cells after AP Induction, and These Effects Were Enhanced when Gln Was Administered Concomitantly with HIF-1*α* Knockdown

**Figure 8:**
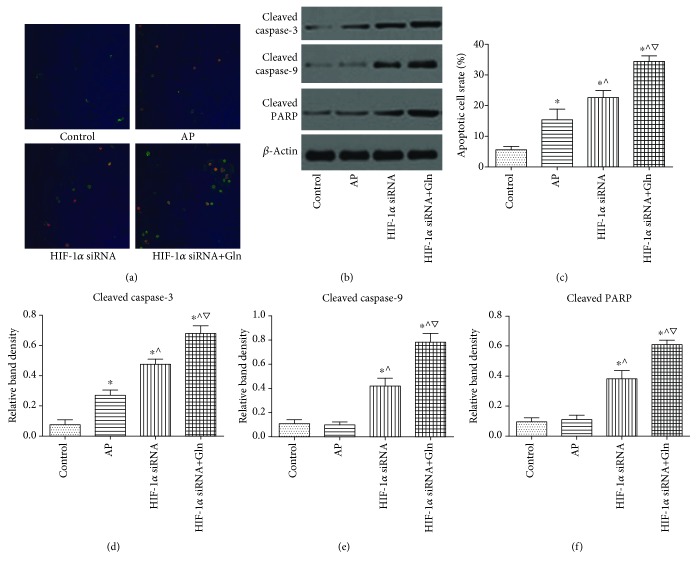
HIF-1*α* knockdown decreased necrosis and increased apoptosis within AR42J cells after AP induction, and these effects were enhanced when Gln was administered concomitantly with HIF-1*α* knockdown. (a and c) Representative fluorescent images (a, 10x) indicative of apoptotic activity by Annexin V and propidium iodide double-staining in AR42J cells as described in Figure 6(a), and the percentage of green cells (c) was calculated to assess the apoptotic cells rate. (b and d-f) Representative immunoblot images (b) and quantifications of cytoplasm cleaved caspase-3 (d), caspase-9 (e), and PARP (f) expression in AR42J cells as described in Figure 6(a). *β*-Actin was used as the protein loading control. Data were presented as mean ± SD (*n* = 3). ^∗^*P* < 0.05*versus* sham, ^^^*P* < 0.05*versus* AP, and ^▽^*P* < 0.05*versus* HIF-1*α* siRNA. Abbreviations: AP: acute pancreatitis; Gln: glutamine; HIF-1*α*: hypoxia-inducible factor-1*α*; SD: standard deviation.

The apoptosis assay was conducted using Annexin V and PI staining. Various levels of apoptosis were identified within AR42J cells after AP induction compared to those in the control group. Figures 8(a) and 8(c) show that HIF-1*α* knockdown before AP induction decreased necrosis and increased apoptosis relative to that caused by AP induction alone. Moreover, HIF-1*α* knockdown plus Gln supplementation before AP induction resulted in decreased necrosis and increased apoptosis compared to that in the HIF-1*α* siRNA group. The cytoplasmic expression of cleaved caspase-3, caspase-9, and PARP, which was evaluated by Western blot, also confirmed the previous findings. The levels of active caspase-3, caspase-9, and PARP in the HIF-1*α* siRNA group were significantly increased than those in the AP group. In addition, the levels of activated caspase-3, caspase-9, and PARP were significantly increased when Gln was administered together with HIF-1*α* knockdown compared to that in the HIF-1*α* siRNA group (Figures 8(b) and 8(d)–8(f)).

## 4. Discussion

AP continues to be a clinical challenge, and no specific therapy has yet been developed. Although there are some common early-phase characteristics such as abnormal activation of pancreatic enzymes within acinar cells, AP might present as a mild self-limited course or even an eventful life-threatening one. Accordingly, the secondary response of the acinar cells to early intracellular damage, namely, the mode of cell death, becomes a critical factor that determines the degree of the inflammatory response and the occurrence and development of subsequent complications in AP [34].

Apoptosis is an active form of programmed cell death that is characterized by an intact cell membrane and the formation of apoptotic bodies. These cells are eventually digested by phagocytes without inducing inflammation or damage to adjacent cells. Necrosis is a passive cell death process that features severe cell membrane rupture accompanied by a massive release of cellular contents, which triggers a strong inflammatory response. In AP, apoptosis might result in the acinar cells being in a relatively stable or “dormant” condition with no further violent inflammation, which might significantly improve AP prognosis. In patients with pancreatitis that causes detectable necrosis in ≥50% of the pancreas, the mortality rate can approach 20% [35]. It has been confirmed that acinar cell apoptosis is a self-protective phenomenon in AP, and inducing acinar cell apoptosis could significantly alleviate the severity of AP and its progression [7, 35]. Our study showed that the HIF-1*α* siRNA+Gln group demonstrated the highest proportion of acinar cell apoptosis and the least damage to the pancreatic tissue, which further helped to verify the significance of acinar cell apoptosis in AP development.

The mitochondrion not only is the major site of cellular oxidative phosphorylation and energy supply but also plays a crucial role in modulating necrosis and apoptosis [36, 37]. Recently, a few reports have emphasized the mitochondrion and its alterations in the pathogenesis of AP [38–40]. Injury-related stress factors associated with AP could induce dysfunction of the Ca^2+^-ATP kinase (Ca^2+^ pump) of the acinar cells, which would eventually lead to intracellular Ca^2+^ overload. This phenomenon is considered to be an early key event in AP and contributes extensively to the exacerbation of its progress [4, 41–45]. Intracellular Ca^2+^ overload triggers the constant Ca^2+^ ingestion of mitochondria [46], which accelerates the loss of the mitochondrial membrane potential due to mitochondrial membrane permeabilization as the result of the opening of the mitochondrial membrane permeability transition pore (PTP). These processes directly induce adverse changes in the mitochondrial structure and function as well as disordered energy metabolism [36, 47–49].

It has been shown that mitochondrial membrane permeabilization is the common onset pathway to cell necrosis and apoptosis, and the intracellular ATP level following mitochondrial membrane permeabilization has been proven to play a key role in determining the death mode of the cells [50]. In our opinion, maintenance of a high ATP level in the impaired acinar cells after AP induction might promote the cellular necrosis-to-apoptosis transformation by the following mechanisms: ① Ca^2+^-ATP kinase is a highly ATP-dependent enzyme system that consumes much energy. ATP could enhance the Ca^2+^-ATP kinase activity of the cell membrane and endoplasmic reticulum, thus resulting in the pumping of the overloaded Ca^2+^ out of the cell or into the endoplasmic reticulum, which would further reduce the intramitochondrial Ca^2+^ level and the degree of PTP opening. When the PTP opening is effectively suppressed, the massive loss of mitochondrial membrane potential and constant depletion of ATP could be reversed, which would allow the cellular energy metabolism balance to recover so that the necrosis is prevented [36]. ② ATP is the trigger for caspase activation which has a cascade amplification effect [32, 35]. The caspase pathway plays a critical role in regulating the cellular necrosis/apoptosis balance. Caspase activation can not only induce cellular apoptosis but also prevent necrosis by inhibiting the activation of polyadenosine diphosphate ribosomal polymerase [7, 32, 35]. However, inactivation of caspase commonly aggravates the cellular necrosis as well as the severity of lesion [35]. Furthermore, chromatin condensation, apoptotic body formation, and other key aspects of apoptosis all require a certain level of ATP [51]. Our results showed that HIF-1*α* siRNA+Gln could decrease the intracellular Ca^2+^ level and the sustained loss of mitochondrial membrane potential by increasing the ATP contents within acinar cells, further increasing the cellular expression of caspase-3 and -9 and PARP, which would promote the transformation of acinar cell necrosis to apoptosis. Recently, accumulating evidence has suggested that the balance between apoptosis and necrosis influences the severity of AP [40, 52]. The promotion of apoptosis ameliorates AP-related injuries and *vice versa* [7, 53]. We therefore considered that the energy metabolism pathway within pancreatic acinar cells might be a promising focus in the pathogenesis of AP.

There are two main aspects to the function of HIF-1*α* [54]. First, HIF-1*α* modulates intracellular glucose metabolism by promoting glycolysis and inhibiting oxidative phosphorylation. Second, HIF-1*α* enhances the tissue oxygen concentration through vasodilation by the induction of inducible nitric oxide synthetase (iNOS) and endothelin-1 (ET-1) and by increasing the erythrocyte concentration through the upregulation of erythropoietin (EPO) [55]. Both our present results and previous findings suggested that the expression of HIF-1*α* was upregulated within acinar cells after AP induction [43, 56]. However, the regulatory effects of HIF-1*α* in AP are poorly understood. In the present study, HIF-1*α* knockdown ameliorated the acinar cell injuries, suppressed the overwhelming inflammatory response, and diminished the necrotic area in the pancreas, but increased the acinar cells apoptotic rate *in vivo*. Moreover, HIF-1*α* knockdown ameliorated the cellular energy metabolism stress and maintained the concentration of Ca^2+^ and the stability of the mitochondria *in vitro*. In general, our results indicated that HIF-1*α* knockdown attenuated the predominance of necrosis over apoptosis after the attack of AP.

Gln is an important energy substance in the body. It has a powerful glycemic effect which can provide sufficient substrates for oxidative phosphorylation of the cells. Gln can be administered to maintain gut integrity and ameliorate the inflammatory response in AP [17–19]. Our results indicated that Gln supplementation in addition to HIF-1*α* knockdown was more protective for the acinar cells from AP-related injuries than HIF-1*α* knockdown alone was. Therefore, the combination of Gln supplementation and HIF-1*α* knockdown might be a promising strategy in the future management of AP.

In this study, the application of HIF-1*α* silencing combined with Gln supplementation could regulate the energy metabolism of the inflammatory acinar cells and promote their necrosis-to-apoptosis transformation, which has a potential clinical value for improving the prognosis of AP. In our opinion, the significance and advantages of this experiment are as follows: ①glycogenic oxidative phosphorylation is the main means by which acinar cells obtain ATP to maintain their physiological function. However, the massive activation of HIF-1*α* during AP significantly inhibits the cellular oxidative phosphorylation of glycogen. Consequently, glycolysis becomes the major pathway for acinar cells to obtain ATP. Glycolysis is a form of energy metabolism with high consumption and low output, which can only maintain the cell energy supply over a limited time course. Continuous and excessive glycolysis leads to the accumulation of its end-product, lactic acid, which causes cellular acidosis and further significant damages to the structure and function of the cells. In addition, the disturbance of the pancreatic microcirculation in AP leads to the lack of adequate supply of glycogen in local pancreatic tissues, which accelerates the depletion of glycogen in acinar cells and eventually induces the depletion of ATP as the result of an increased anaerobic glycolysis capacity associated with “no glucose for glycolysis.” Therefore, it is not the best compensatory mechanism to regulate energy metabolism through enhancing glycolysis of the acinar cells by activated HIF-1*α*. Inhibiting the activation of HIF-1*α* to avoid the waste of glycogen and permit the limited glycogen supply to undergo oxidative phosphorylation may turn out to be more favorable by allowing the acinar cells to continuously produce enough ATP and thus accelerate the necrosis-to-apoptosis transformation. ② HIF-1*α* can not only inhibit the mitochondrial tricarboxylic acid cycle by activating pyruvate dehydrogenase kinase-1 [30] but also suppress the mitochondrial respiratory function by upregulating iNOS to induce massive synthesis of NO [9, 57]. Therefore, inhibition of HIF-1*α* activation could allow the full use of a limited oxygen supply to enhance mitochondrial oxidative phosphorylation which would result in the production of sufficient ATP. ③ Downregulation of HIF-1*α* expression could effectively improve pancreatic microcirculatory dysfunction by suppressing the synthesis of EPO and ET-1, which would increase the glycogen supply and oxygen content in acinar cells with the results that the mitochondrial oxidative phosphorylation process would be enhanced. ④ Traditional apoptosis inducers are nonselective and might simultaneously induce apoptosis of both damaged and normal acinar cells. Clearly, inducing normal acinar cell apoptosis would further impair the physiological function of the pancreas and aggravate AP. In this study, on the one hand, the acinar cells in which we increased the ATP contents by the combination of HIF-1*α* silencing and Gln supplementation were mainly the injured acinar cells, and this treatment had no harmful effect on the normal cells. Therefore, this therapy could protect the physiological function of the pancreas to the maximum extent by endowing the apoptosis induction with certain selectivity, thus avoiding excessive apoptosis and extra damage to the pancreas. On the other hand, obtaining sufficient ATP in the normal acinar cells is an important guarantee to maintain their physiological function, which can enhance their ability to resist inflammatory attack. Therefore, the design of this study might achieve the dual effects of “treatment and prevention.”

## 5. Conclusion

In conclusion, the present study highlights the promising effects of Gln-supplemented HIF-1*α* knockdown in the management of AP. The underlying mechanisms may be attributed to the amelioration of intracellular energy stress through the maintenance of mitochondrial homeostasis and prevention of intracellular Ca^2+^ overload, which result in the attenuation of the predominance of necrosis over apoptosis.

## Data Availability

The data used to support the findings of this study are included within the article.
